# High-performance asymmetric supercapacitor based on CdCO_3_/CdO/Co_3_O_4_ composite supported on Ni foam†

**DOI:** 10.1039/d1ra05243h

**Published:** 2021-09-23

**Authors:** Rodrigo Henríquez, Alifhers S. Mestra-Acosta, Eduardo Muñoz, Paula Grez, Elena Navarrete-Astorga, Enrique A. Dalchiele

**Affiliations:** Instituto de Química, Facultad de Ciencias, Pontificia Universidad Católica de Valparaíso Casilla 4059 Valparaíso Chile rodrigo.henriquez@pucv.cl +56 32 2274921; Universidad de Málaga, Departamento de Física Aplicada I, Laboratorio de Materiales y Superficies (Unidad asociada al CSIC) E29071 Málaga Spain; Instituto de Física, Facultad de Ingeniería Herrera y Reissig 565, C. C. 30 11000 Montevideo Uruguay

## Abstract

A CdCO_3_/CdO/Co_3_O_4_ composite has been prepared on nickel foam through a combined hydrothermal-annealing method. An asymmetric hybrid supercapacitor (SC) device was assembled with this composite as the positive electrode and activated carbon was the negative electrode. The SC exhibited a high specific capacitance of 84 F g^−1^ @ 1 mA cm^−2^, a maximum energy density of 26.3 W h kg^−1^, and a power density of 2290 W kg^−1^, along with a wide potential window of 1.5 V and long cycle life (92% after 6000 cycles). SCs assembled in series powered various light-emitting diodes and moved an electrical mini-motor.

## Abstract 2

This work presents for the first time a CdCO_3_/CdO/Co_3_O_4_@nickel foam based supercapacitor with high both specific capacitance and energy density, a widespread potential window and a long cycle life.

## Graphical abstract

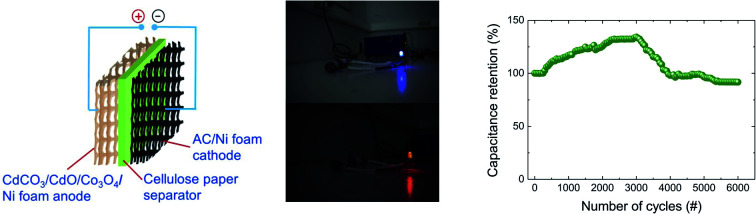

## 1 Introduction

The rapid development of civilization and the high rate of population growth has resulted in the increased consumption of resources and, therefore, the demand for energy in the 21^st^ century, which has caused enormous research efforts related to energy conversion and storage.^1,2^ As such, better energy storage devices are in urgent demand.^3^ Of the electrochemical energy storage devices, supercapacitors (SCs) are known as the most promising due to their high power delivery density, rapid charge–discharge rate, long cycle life, and safety of operation.^4–6^ However, their low energy density is considered a major challenge since it restricts their applications.^4^

SCs are divided into electric double-layer capacitors (EDLCs) and pseudocapacitors.^3,7^ The EDLCs rely on the physical adsorption and accumulation of electrostatic charges at the electrode/electrolyte interface, while the pseudocapacitors utilize the reversible valence changes in electrode materials to store/release energy generated by redox reactions.^3,7^ In recent years, the combined advantages of capacitive (for a high power density) and faradaic (for a high energy density) mechanisms in one supercapacitor device have led to the coining of the term “hybrid supercapacitor”.^3,7^ Hybrid capacitors are the combination of an EDLC and a pseudocapacitor electrode in one SC.^3,7^ Asymmetric SCs (ASCs) assembled using two dissimilar electrode materials in an aqueous electrolyte are a practicable way to improve the energy density by widening the operation voltage window.^7^ The fabrication of asymmetric/hybrid SCs is a promising approach for overcoming the shortfalls of a low energy density in supercapacitors.^8^

The performance of SCs is dependent on the intrinsic properties of active electrode materials.^9^ Electrochemical double-layer capacitors (EDLC) employ mainly carbon derivatives materials with high surface area per unit, such as activated carbon, carbon nanofibers, or carbon nanotubes.^10^ On the other hand, pseudocapacitors use hydroxides or metallic oxides, such as manganese oxide (MnO_2_),^11^ nickel oxide (NiO),^12^ cobalt hydroxide Co(OH)_2_,^13^ ruthenium oxide (RuO_2_),^14^ ferric oxide (Fe_2_O_3_),^15^ and conducting polymers or copolymers such as polyaniline (PANI), polypyrrole (PPy), or a combination of these.^16^ Electrodes composed of cobalt, cobalt oxide^17,18^ and these hybridized with property-complementary nanomaterials, *i.e.*, metals,^18^ carbon-based materials,^19^ sulfides, or conducting polymers^20^ have provided high specific capacitances and excellent cyclability as materials in SCs. Another metal used in SCs is cadmium (Cd), providing capacitance values of 1190 mF g^−1^ when used as CdO.^21^ Moreover, it has been demonstrated that the specific capacitance of cobalt electrodes was improved when doped or mixed with cadmium to obtain a specific capacitance of 737 F g^−1^ at a current density of 1 A g^−1^.^22^ In the last few years, studies were conducted on the CO_3_^2−^ ion with transition metals in many electrochemical storage applications, such as manganese carbonate (MnCO_3_) with a capacitance of 216 F g^−1^ in SCs, improving wettability and electrode stability,^23^ and CoCO_3_ in Li-ion batteries demonstrating better stability than Co_3_O_4_.^24^ Very recently, hydroxycarbonates with M_2_(CO_3_)(OH)_2_ stoichiometry (where M represents the metal cations of Ni, Fe, Cu, Co, Zn, *etc.*) have received considerable attention as electro-active materials for high-performance supercapacitors due to high theoretical capacitance and variable compositions. For instance, electrode materials, such as the MoS_2_/NiCo(OH)_2_CO_3_ composite (as a nanosheet-assembled 3D flower-like nanostructure),^25^ nickel–cobalt–manganese ternary carbonate hydroxide (as ultrathin nanoflakes)^26^ and dendritic Co_3_O_4_@Co_2_(CO_3_)(OH)_2_ nano-arrays,^27^ have been reported to give high-performance energy storage devices.

Moreover, it has also been reported that electrodes composed of 3D structures integrated with nickel foam (NF) as the current collector, with a porous architecture design without additives or binders provide high specific capacitance values.^2,7^

In this work, an asymmetric hybrid SC with high energy density was designed from a mixture of Co_3_O_4_/CdO/CdCO_3_@NF by means of direct growth without additives or binders on an NF substrate.

## 2 Experimental methods

### 2.1 Materials

Commercial nickel foam (thickness: 1.1 mm, volume density: 0.45 g cm^−3^, average pore diameter: 13.1 mm, PPI: 95–110, porosity: 95%, specific surface areal density: 330 ± 10 g m^−2^) was supplied by Career Henan Chemical Co. Ltd. (China). Cobalt nitrate hexahydrate (Co(NO_3_)_2_·6H_2_O, ≥98.8%), cadmium nitrate hexahydrate (Cd(NO_3_)_2_·6H_2_O, ≥99%), urea (≥99.5%), and sodium hydroxide (KOH, ≥90%) were purchased from Sigma-Aldrich (USA). Activated carbon (AC) (≥99.5%), polyvinyl alcohol (PVA) (99%). Carbon nanotubes (CNT) were synthesized by the chemical vapor deposition technique (CVD) in a tube furnace, using a temperature of 750 °C and C_2_H_4_ gas as raw material and carbon source, without additional catalysts. A cellulose paper (Merck) was used. All chemicals were used as received without further purification. Deionized (DI) water obtained through a Millipore system (Milli-Q) with a resistance of ∼18 MΩ cm^−1^ was used in the reactions.

### 2.2 Synthesis of the CdCO_3_/CdO/Co_3_O_4_@NF electrode

The active composite material (CdCO_3_/CdO/Co_3_O_4_) was synthesized directly on the nickel foam substrate through a hydrothermal-annealing process. First, the nickel foam was cut into disk-shaped pieces with a diameter of 1.31 cm (1.35 cm^2^ of geometrical area). The foam nickel samples were washed in acetone for 10 min, and then etched in 1 M HCl for 10 min to remove the surface oxide layer, and finally rinsed several times with DI water. Further, in detail, 2 mmol of Co(NO_3_)_2_·6H_2_O, 2 mmol of Cd (NO_3_)_2_·6H_2_O, and 15 mmol of urea were mixed in 50 ml of deionized water; after 10 minutes of stirring, the obtained homogeneous reaction solution was transferred along with a piece of clean nickel foam into a Teflon-lined stainless steel reactor. It was sealed and maintained at 95 °C for 8 h, and then allowed to cool to room temperature. The prepared sample was collected and rinsed with DI water several times. Then, the active material supported on the nickel foam was subjected to heat treatment at 450 °C under an argon flow of 105 sccm for 3 h, to obtain the composite electrode. The resulting active material mass loaded onto the NF was about 8 mg.

### 2.3 Synthesis of the activated carbon@NF negative electrode

The preparation of the negative electrode has been carried out following a modified technique that had previously been reported.^28^ The active material (AC) was mixed with CNT and PVA in the weight ratio of 80 : 5 : 15, respectively, using ethanol as a solvent to form a homogeneous slurry. First, the AC was mixed with the CNTs in an agar mortar until a homogeneous mixture was obtained, then the PVA and 1 ml of ethanol were added to this mixture to obtain the carbon-based slurry. Further, the 1.31 cm diameter nickel foam sample was dipped into this carbon ink for 1 hour. Subsequently, to evaporate the solvent, the electrode was dried at 78 °C for 24 hours in an oven in air. The resulting electrode had a geometric surface area of 1.35 cm^2^ and exhibited a mass of active material of 15 mg.

### 2.4 Morphological, structural, and surface chemistry characterization

The structural characterization of the composite material was carried out by X-ray diffraction (XRD) on a PANalytical X'Pert Pro automated diffractometer. Patterns were recorded in the Bragg–Brentano configuration using a monochromatic high intensity (Cu K_α1_) radiation. An X'Celerator detector with a step size of 0.017° (2*θ*) was used, and the working power was 45 kV × 40 mA. Field emission scanning electron microscopy (FE-SEM) images of the electrodes were obtained on a Helios Nanolab 650 Dual Beam equipment from FEI Company. The analysis of the chemical composition of the formed structures was carried out using X-ray energy dispersion spectrometry (EDS). The equipment used was a QUANTAX 200 model from Bruker with XFLASH (EDX coupled to SEM equipment: Hitachi SEM SU-3500 of variable pressure with a detector 410-M). Samples for TEM were prepared by removing the gridded nanostructured grown material with a scalpel, then they were collected and ultrasonically dispersed in 1 ml of ethanol. A small drop of the suspended solution was placed on a porous carbon film on a nickel screen and allowed to air dry. Transmission electron microscopy (TEM) and high-resolution transmission electron microscopy (HRTEM) images were obtained on a Talos F200X instrument.

The oxidation states of the chemical elements were studied *via* X-ray photoelectron spectroscopy (XPS) using an ESCA 5701 from Physical Electronics (PHI). An Mg K_α_ radiation source (15 eV) with an operating power of 400 W in an ultra-high vacuum system at a base pressure of ∼1.3 × 10^−8^ Pa was employed.

### 2.5 Assembly of the asymmetric hybrid supercapacitor

The supercapacitor was built using a two-electrode Swagelok cell, with a CdCO_3_/CdO/Co_3_O_4_@NF disk as the positive electrode, a cellulose paper as a separator (3 M KOH as the electrolyte), and an AC@NF disk as the negative electrode, all these parts were sandwiched and mounted into the cell holder between two stainless steel electrodes and compressing by a spring.

### 2.6 Electrochemical characterization

The electrochemical behavior of the resulting supercapacitor was evaluated by cyclic voltammetry (CV), galvanostatic charge–discharge (GCD) measurements and electrochemical impedance spectroscopy (EIS) carried out at room temperature with an electrochemical workstation (PGSTAT 30 AUTOLAB). EIS measurements were performed by applying an AC voltage with 10 mV amplitude in a frequency range from 0.1 Hz to 100 kHz at open circuit potential. Equivalent circuit modeling of the EIS spectrum has been analyzed by the ZView Software.

The specific capacitance per unit mass *C*_m_ (F g^−1^), energy density *E* (W h kg^−1^), and power density *P* (W kg^−1^) of the SC determined from the galvanostatic charge/discharge data were given by the following:^3,25^1
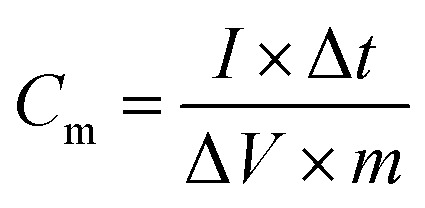
2
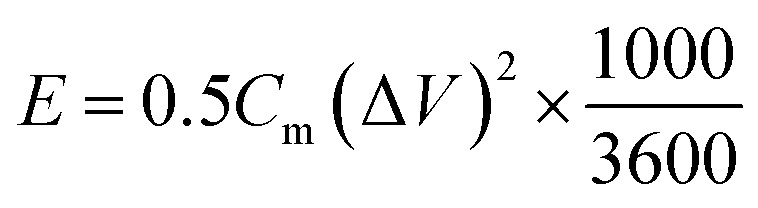
3
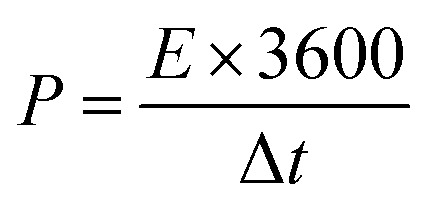
where *I* (A) is the applied current, Δ*t* (s) is the discharge time, Δ*V* (V) is the operating voltage window (excluding the *IR* drop) and *m* (g) is the mass of the active material.

The mass matching of the positive and negative electrodes, respectively, was decided using the capacitor charge balance principle (*Q*^+^ = *Q*^−^) in the steady-state as follows:^29^4
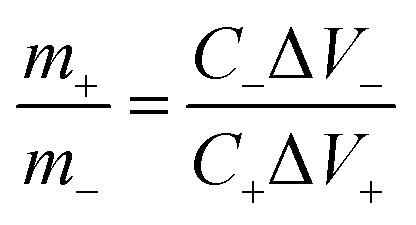
where *C* and *m* are the specific capacitance and the mass of electro-active materials, respectively, and Δ*V* is the applied voltage window for the negative electrodes (−), as well the positive ones (+).

## 3 Results and discussion

**Fig. 1:**
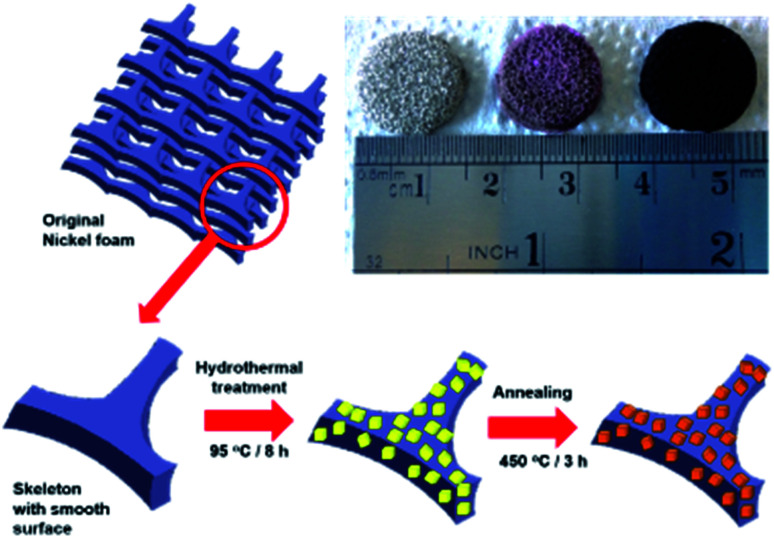
Schematic illustration of the preparation process of the CdCO_3_/CdO/Co_3_O_4_ composite on a 3D nickel foam electrode. The inset shows a digital photograph of a bare nickel foam sample (left), the sample after the hydrothermal step (center), and the resulting composite electrode after the annealing process.

The representative synthetic procedure of the CdCO_3_/CdO/Co_3_O_4_@NF hybrid electrode is depicted in Fig. 1 The inset of Fig. 1 shows digital photographs of pristine NF and composite@NF samples after the hydrothermal process and after the annealing step. The color changed from gray for NF, to an intermediate magenta color, to black for the resulting SC electrode.

### 3.1 Morphological and structural properties of the CdCO_3_/CdO/Co_3_O_4_@Ni foam electrode

**Fig. 2:**
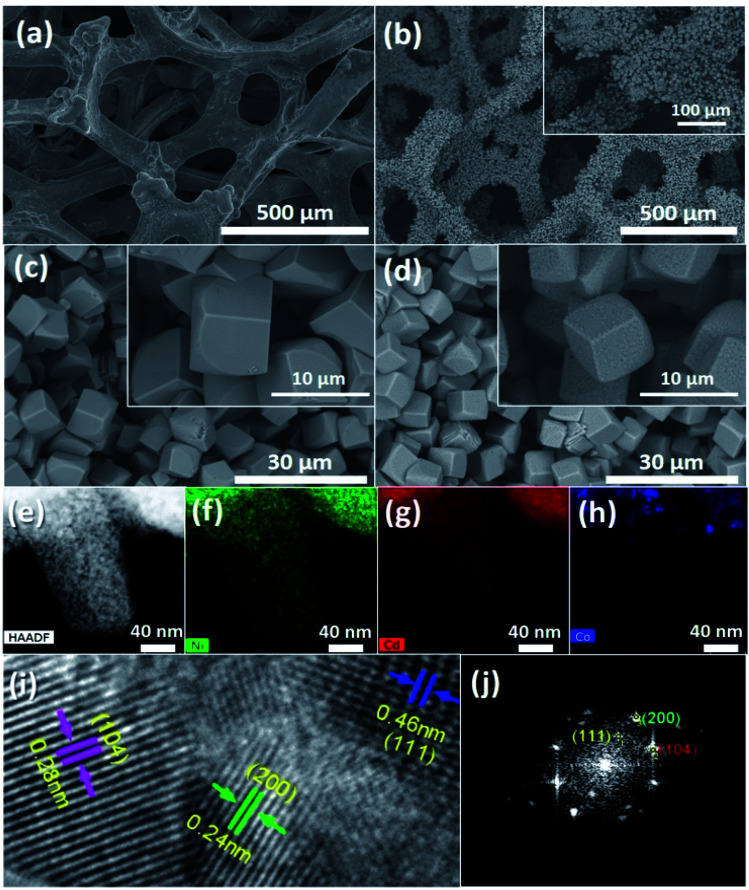
SEM plane-view micrograph images of (a) the pristine nickel foam substrate and (b) the nickel foam after the hydrothermal process. SEM micrograph images depicting in more detail the cubic architectures exhibited by the CdCO_3_/CdO/Co_3_O_4_ composites on nickel foam (c) after the hydrothermal process and (d) after an additional annealing treatment. The insets show high magnifications SEM micrographs. (e) TEM image of a nano-portion of nickel foam covered by the composite under study and the corresponding EDX elemental mapping of Ni (green), Cd (red) and Co (blue), depicted in panels (f), (g) and (h), respectively. (i) HRTEM micrograph of a CdCO_3_/CdO/Co_3_O_4_ crystallite, lattice fringes corresponding to the involved chemical compounds are indicated; the corresponding FFT pattern is depicted in panel (j).

The morphological properties of bare nickel foam and CdCO_3_/CdO/Co_3_O_4_@Ni foam samples have been investigated by SEM observations as displayed in Fig. 2. An SEM image of the nickel foam substrate is depicted in Fig. 2a, showing a 3D porous network structure with pore size diameters from 150 to 370 μm, supplying a large surface area for supporting active materials. Fig. 2b depicts SEM micrograph images of a sample that underwent the hydrothermal process, showing that the composite densely and uniformly covers the skeleton surface of the nickel foam, with a high loading mass per NF real surface area of 4 mg cm^−2^. It must be pointed out that this very good coverage is maintained even after the high-temperature annealing step. The composite material remained well adhered to the Ni foam substrate after being rinsed under centrifugation at a speed of 500 rpm for two minutes, indicating that it is an effective and robust binder-free integration. Fig. 2c and d show high magnification SEM micrographs of samples that underwent hydrothermal and hydrothermal plus high-temperature post-annealing processes, respectively. It can be seen that the composite consists of an agglomeration of micro-particles with cubic shapes, with an edge size of about 5–8 μm. The samples obtained after the hydrothermal process exhibited cubic particles with smooth surface faces (see Fig. 2c). However, in the samples that experienced hydrothermal and high-temperature post-annealing processes, the presence of roughness and nano-pores on the faces of the composite cubic micro-particles can be seen (see Fig. 2d). The presence of these nano-pores can be due to the evolution of the CO_2_ product of the chemical decomposition of previously formed cadmium carbonate and the dehydration of cobalt hydroxide; both compounds were formed during the hydrothermal step process.

Fig. 2e shows a TEM image of a nano-portion of the CdCO_3_/CdO/Co_3_O_4_@Ni foam sample; Fig. 2f–h depict the corresponding EDS elemental mapping, revealing the presence of Ni, Cd, and Co elements in the sample. The high-resolution TEM image depicted in Fig. 2i (obtained from the zone indicated by a dashed rectangle in Fig. S1†), reveals three lattice fringes of 0.28, 0.24, and 0.46 nm, corresponding to the (104), (200), and (111) lattice planes of the rhombohedral phase of CdCO_3_, the cubic phase of CdO and the cubic phase of Co_3_O_4_ (see ESI†). Fig. 2j depicts the corresponding fast Fourier transform (FFT) pattern of this HRTEM result. The very clear crystal lattice as well the corresponding well-ordered dot pattern of the FFT image demonstrated the high-quality single-crystalline nature of the composite. However, structural analysis through XRD of the composite samples revealed only diffraction peaks corresponding to CdCO_3_ and CdO phases, and those originated from the Ni substrate, and the lack of those corresponding to the Co_3_O_4_ phase (see Fig. S2 and S3†). EDS microanalysis results (see Fig. S4 and Table S1†) showed high oxygen and cadmium contents and low cobalt content. It must be pointed out that X-ray diffraction statistically gives us a good idea of the average sample, whereas electron diffraction in TEM allows us to obtain local structure information. It was inferred that there was a low quantity of the Co_3_O_4_ phase, which was formed by grains with small crystallite sizes, evidencing a low degree of crystallinity. XPS photoelectron spectroscopy analysis confirmed the presence of those compounds, see Fig. S5.†

### 3.2 Supercapacitor electrochemical performance

**Fig. 3:**
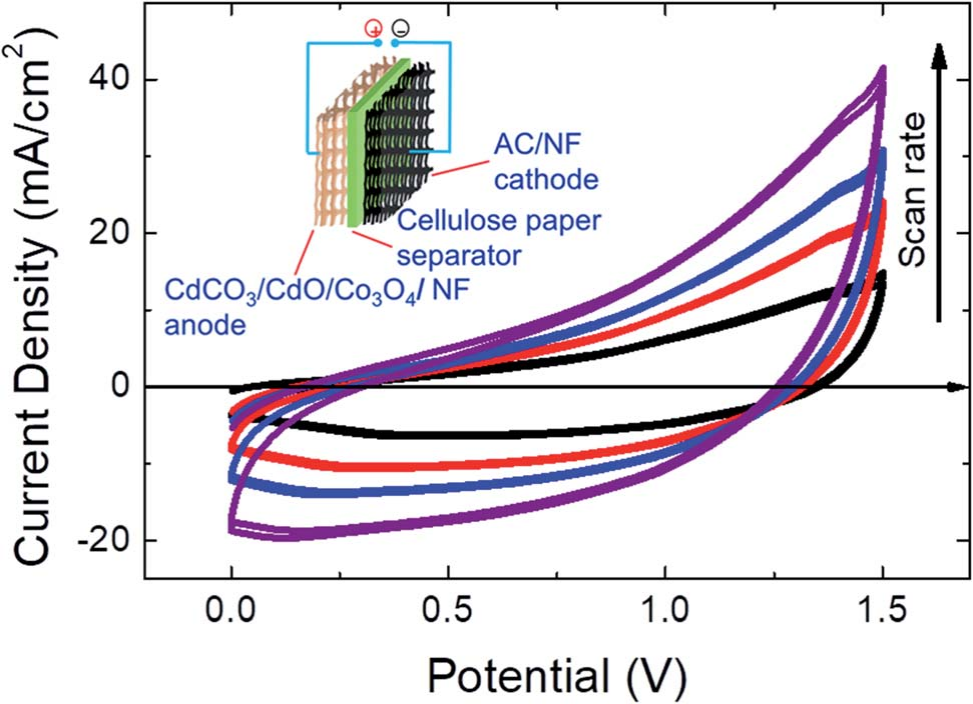
Cyclic voltammetry of the CdCO_3_/CdO/Co_3_O_4_@Ni//AC supercapacitor device at different potential scan rates (10, 20, 30, and 40 mV s^−1^). Inset: schematic diagram of the asymmetric supercapacitor configuration.

To further evaluate the CdCO_3_/CdO/Co_3_O_4_@Ni foam hybrid electrode for real application, a 1.35 cm^2^ ASSC device was made by using the CdCO_3_/CdO/Co_3_O_4_@Ni electrode as the positive one and the activated carbon (AC) film on Ni foam as the negative one in 3 M KOH with one piece of cellulose paper as the separator, (working potential is 1.5 V, see Fig. S6†), *i.e.*: the CdCO_3_/CdO/Co_3_O_4_@Ni//AC SC device. In the inset of Fig. 3, a scheme of the SC developed in this work shows the different components. To optimize the quantity of charge balance between positive and negative electrodes, the loading mass ratio between the CdCO_3_/CdO/Co_3_O_4_ composite and AC electrodes was estimated by using eqn (4). According to this formula, the optimized mass ratio is ∼0.43. The mass values finally obtained were 8 mg and 15 mg for the positive and negative electrodes, respectively, leading to an optimized mass ratio of ∼0.53. The electrochemical performance of this ASSC was investigated *via* two-electrode measurements. The cyclic voltammetric (CV) curves at various scan rates of the ASSC are shown in Fig. 3. The device exhibited a quasi-rectangular CV shape. Even at high scan rates, the CV curves do not show obvious polarization, confirming that the cell potential of 1.5 V is reasonable.^5^ With the increasing scan rate, the shape of the CV curves remained unaltered and an increase of the device current was observed, which demonstrated the high rate capability of the device.^6^ This is indicative of an effective electric transport of ions onto the electrode surface. The large area under the curves revealed high capacitance values, as shown in Table S2,† for scan rates from 5 to 50 mV s^−1^. Because of the mutual effect of the positive and negative electrodes, the operation potential window could reach as high as 1.5 V. The potential window exhibited by this SC is higher than that observed in conventional symmetric SCs based on AC electrodes in aqueous electrolytes (0.8–1.0 V),^7,8^ and higher than reported SC potential windows of SCs based in cobalt compounds (1.4 V)^30^ and cadmium compounds (*ca.* 1.4 V)^31^ active electrode materials. Indeed, this high operation potential window leads to the achievement of high-energy storage density values as will be discussed later. One of the key criteria for the performance of an SC is the self-discharge characteristics.^8^ Self-discharge is the spontaneous decrease in the voltage of the charged SCs with time under open-circuit conditions. It is very important to suppress this phenomenon, which will enable SCs to store energy more efficiently.^8^ The CdCO_3_/CdO/Co_3_O_4_@Ni//AC SC device showed a self-discharging rate (final voltage/initial voltage) of *ca.* 13.5% after 22 hours (see Fig. S7†).

**Fig. 4:**
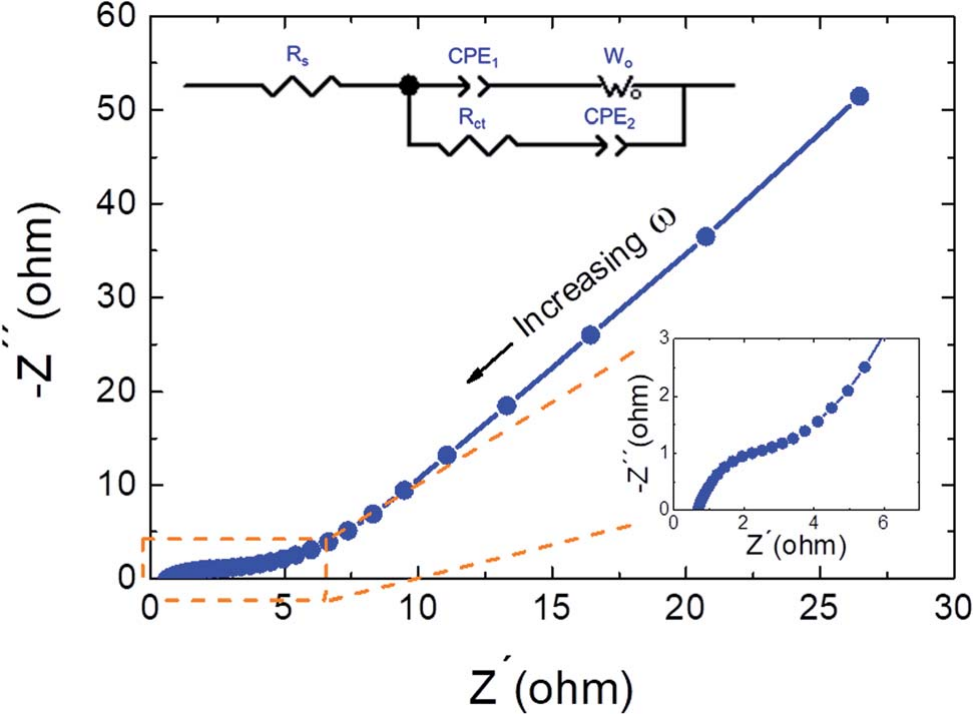
Nyquist plot for the CdCO_3_/CdO/Co_3_O_4_@Ni//AC supercapacitor device at open potential conditions. The symbols (blue circles shapes) represent the experimental data; the blue line is the fitting result with the electrical circuit model. The upper inset shows the equivalent electronic circuit model used to fit the experimental EIS data, whereas the lower one shows an enlargement of the plot in the high-frequency range.

The electrochemical performance and the electrochemical events occurring within the electrodes of the supercapacitor were further investigated using electrochemical impedance spectroscopy (EIS) analysis. EIS measurements for the CdCO_3_/CdO/Co_3_O_4_@Ni//AC SC devices have been performed using a sinusoidal signal in the frequency range from 0.1 Hz to 100 kHz at open circuit potential, and the corresponding Nyquist plot for a typical SC sample is shown in Fig. 4. A nearly straight line was observed in the low-frequency range and a small semicircle in the high-frequency region. The straight line represents the Warburg impedance, which indicates the diffusive impedance of electrolyte ions in the CdCO_3_/CdO/Co_3_O_4_ composite (diffusive resistance of electrolyte ions in the interior cavities of the electrode), and the semicircle diameter of EIS is assigned to the charge-transfer resistance (*R*_ct_), while the intersection between the curve and the *x*-axis indicates the resistance of equivalent series (*R*_s_).^5,25,29,32,33^ Moreover, that small semicircle indicates both the pseudocapacitive nature of the composite material and higher charge transfer rates.^34^ The Nyquist plots were then fitted with an equivalent electrical circuit (see inset of Fig. 4) and the interpreted data are shown in Table 1. The fit indicated by a solid line in the plot showed good agreement with the data. This confirmed the suitability of the equivalent circuit model used. The total SC impedance can be accurately modeled as serial and parallel combinations of different circuit components (see inset of Fig. 4): a serial resistance *R*_s_ denoting the electrolyte solution resistance and a parallel combination constituted by *R*_ct_ charge transfer resistance, constant phase shift elements (CPE_1_ and CPE_2_) and a Warburg impedance (*W*_o_). An electrolyte solution serial resistance *R*_s_ = 0.62 Ω was obtained (see Table 1), indicating the very good quality of the interfacial contact between the composite electrode and the electrolyte. The *R*_ct_ charge transfer resistance exhibited a value of 4.32 Ω as can be seen in Table 1. Low *R*_s_ and *R*_ct_ values indicate the low internal resistance, good ion accessibility, rapid faradaic redox reactions, and high electrochemical reversibility in the low-frequency region due to the nano-structure of the composite.^32^ Moreover, as a result of the EIS simulation fitting process, two CPE parameters were obtained: a constant-phase element CPE_1_ to simulate double-layer capacitance (AC@NF electrode interface), and a pseudo-capacitance CPE_2_ of the material redox process (CdCO_3_/CdO/Co_3_O_4_@NF electrode interface).^26^ The corresponding CPE parameters (*Q* constant phase element with power *α*, see note of Table 1),^35^ were extracted. A Warburg impedance element (or diffusion constant) appeared in the equivalent circuit, providing information about the ionic diffusion within the SC.^25^ The Warburg *W*_o_ impedance is a function of three parameters: *W*–*R*, the Warburg diffusion impedance; *W*–*T*, the diffusion time constant, and *W*–*P*, the phase factor (0 < *W*–*P* < 1).^36^ The values achieved by these Warburg parameters (see Table 1), indicate short ionic routes and good capacitive efficiency, similar to that exhibited by other metallic carbonate-based supercapacitors.^25^ The *W*–*P* value of 0.9 indicates a material with a high degree of porosity.^25^

**Table 1:** Equivalent circuit element values obtained through a complex non-linear least-squares fitting of the EIS spectra^a^

*R*_s_ (Ω)	*Q*_1_ b (Ss^*α*1^)	*α*1	*R*_ct_ (Ω)	*Q*_2_ b (Ss^*α*2^)	*α*2	*W*–*R* (Ω)	*W*–*T* (s)	*W*–*P*
0.62	0.0028	0.60	4.32	0.025	0.64	24.07	0.18	0.9

a See inset of Fig. 4 for the involved electrical equivalent circuit.

b The impedance of a CPE is defined as 
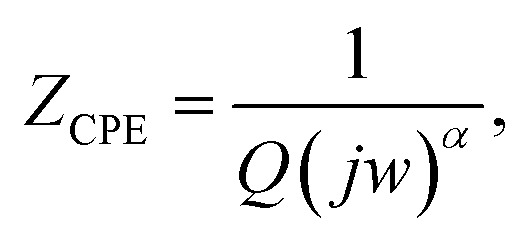
 where *α* (0 < *α* < 1) is an empirical constant with no real physical meaning.

**Fig. 5:**
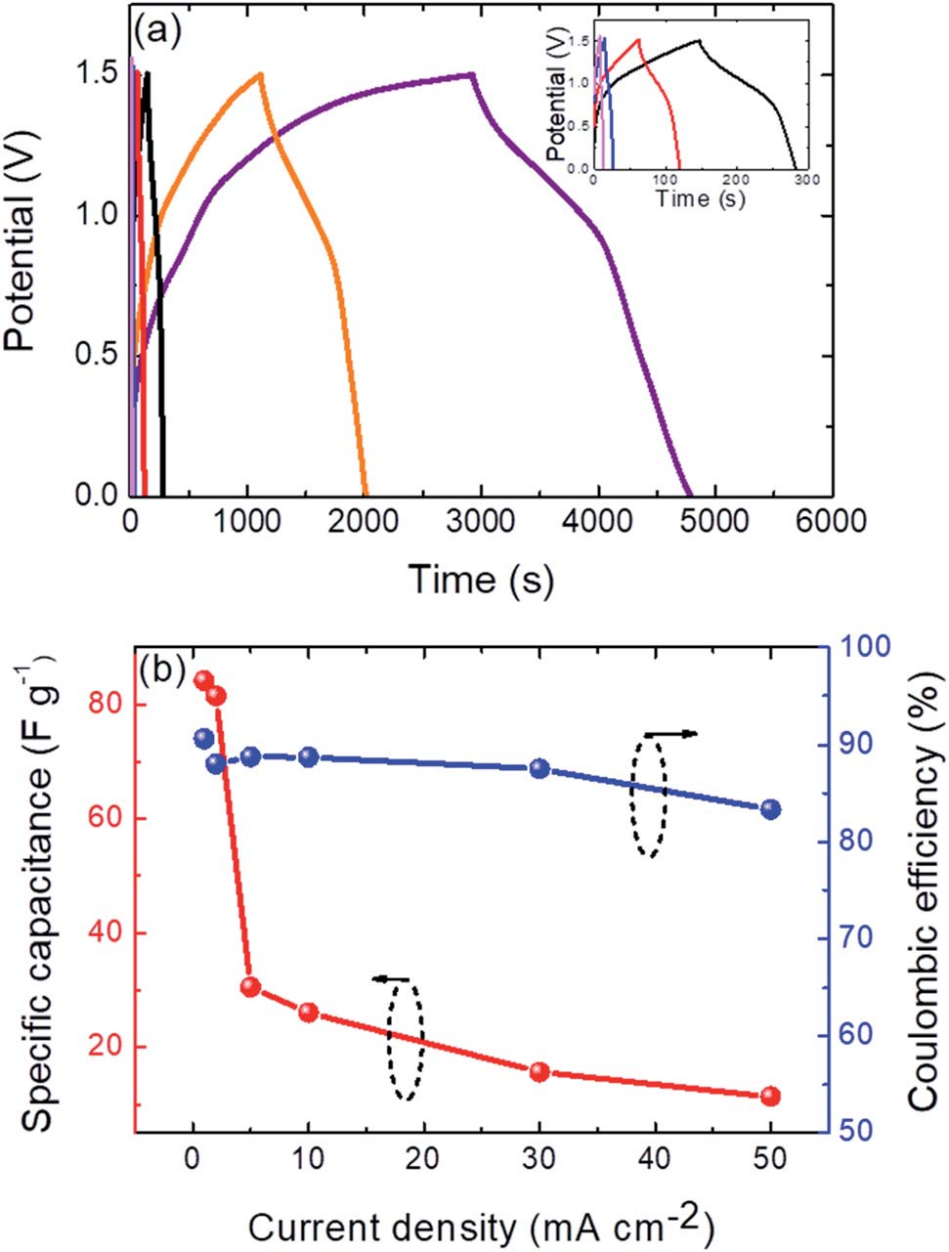
(a) Galvanostatic charge and discharge of the CdCO_3_/CdO/Co_3_O_4_@Ni//AC supercapacitor device at different current densities: 1 mA cm^−2^ (purple line), 2 mA cm^−2^ (orange line), 5 mA cm^−2^ (black line), 10 mA cm^−2^ (red line), 30 mA cm^−2^ (blue line) and 50 mA cm^−2^ (light magenta line). For clarity, the curves corresponding to the highest current density values are depicted in the inset. (b) The rate capability and the coulombic efficiency of the CdCO_3_/CdO/Co_3_O_4_@Ni//AC supercapacitor device at different current densities.

The electrochemical device performance of the SC was evaluated by galvanostatic charge/discharge (GCD) analysis (at various current densities and an operating potential window from 0 to 1.5 V), as illustrated in Fig. 5a. The nearly symmetric GCD curves demonstrated the good reversibility of the charge–discharge process.^37^ The GCD curves did not show the plateaus that are characteristic of a battery, nor were they totally linear, which confirmed the pseudocapacitance characteristics of the compound, indicating that we are facing a hybrid SC, which is consistent with the CV curves that show the absence of redox peaks (see Fig. 3). On increasing the imposed current density value (see inset of Fig. 5a), the GCD curves continued to show a relative symmetrical profile, demonstrating the good reversibility of the charge–discharge process and low polarization of the active material, thus giving high specific capacitance values. This could be due to the high specific surface area exhibited by the cubic micro-particles of the CdCO_3_/CdO/Co_3_O_4_ composite electrode (see Fig. 2). This micro-structure allows the electrolyte to go into the nanopores (formed between the cubic micro-particles), leading to a greater accumulation of electrical charge and improved electrical transport due to the interconnectivity that exists between the micro-cubes. The obtained specific capacitance value (excluding the *IR* drop) is *ca.* 84 F g^−1^ @ 1 mA cm^−2^ GCD current density, which is comparable to, or even higher than recent reports for other ASSCs, such as CuO//AC (72.4 F g^−1^),^38^ MnO_2_//graphite (72.7 F g^−1^)^39^ and Co_3_O_4_//AC (57.4 F g^−1^).^40^ Detailed specific capacitance values at other different GCD current densities are depicted in Table S3.† This obtained high capacitance value can be explained by a synergetic interaction between the CdCO_3_, CdO, and Co_3_O_4_ single phases of the studied composite, leading to an increase in the specific capacitance of the only CdCO_3_ single-phase (results of an exhaustive study of this synergetic effect on the final performance of the composite SC device are now being prepared and will be published). Fig. 5b shows the rate discharge capability of the CdCO_3_/CdO/Co_3_O_4_@Ni//AC supercapacitor device at different current densities. It can be seen that the specific capacitance decreases with the increase in the current density. This is in line with the general law of the rate performance of electrochemical energy storage devices and may be associated with ion-exchange processes.^32,41,42^ It is difficult for the diffusion of ions is to reach the entire electrode material at high current density values.^32,41,42^Fig. 5b also shows the coulombic efficiency of the SC device as a function of the current density. There was a slight decrease in this coulombic efficiency from *ca.* 90% to *ca.* 85% at low and high current density values, respectively, indicating that the reversibility of the charge–discharge process decreases with the increase of the current density values.

As is known, the performance of an SC is dependent on the intrinsic properties of active electrode materials.^9^ In the present case, the specific capacitance of the CdCO_3_/CdO/Co_3_O_4_@Ni//AC SC device (being higher than those exhibited by conventional AC-based symmetric SCs, *i.e.* ∼20 F g^−1^), is on the one hand restricted by the AC negative electrode (∼165 F g^−1^), and in the other hand is significantly improved by the pseudocapacitance of the CdCO_3_/CdO/Co_3_O_4_@Ni electrode (1057 F g^−1^ @ 1 mA cm^−2^ GCD current density, see Table S3† for the capacitance value at other different GCD current density values).

**Fig. 6:**
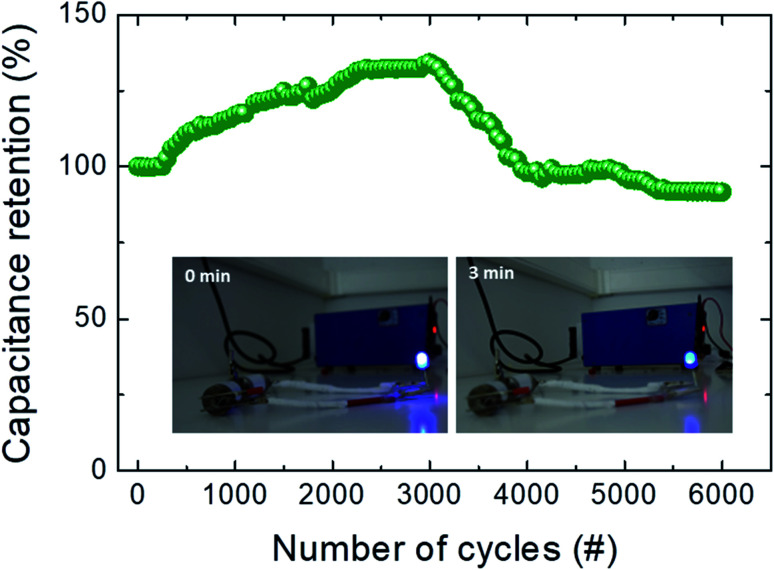
Cycling stability of our device after 6000 cycles from an initial 84.0 F g^−1^ specific capacitance value. Inset: pictures showing that two SCs in series can light a blue LED indicator; the images show different stages powered by the SCs charged for 10 s.

One of the most crucial factors for the practical application of SC devices is long cycle life. Therefore, the cycling stability test was conducted with the hybrid SC device at a current density of 30 mA cm^−2^, and the obtained plot is displayed in Fig. 6. The hybrid SC demonstrated excellent cycling stability (from an initial specific capacitance of 84.0 F g^−1^), with a capacitance retention of *ca.* 92% after 6000 continuous charge–discharge cycles. The capacitance retention increased after the 200 cycles (as can be seen in Fig. 2c), which is likely due to an “activation process”.^2,7,43^ Further, from the 4000^th^ cycle to the 6000^th^ one, a slight decrease in the capacitance retention can be seen, arriving at *ca.* 92% at the end of the GCD cycling. The electrochemical charge and discharge processes can generate crystal imperfections at the level of the composite cubic-shaped micro-particles, and then a higher porosity of the composite electrode can be achieved. This allows the diffusion of OH^−^ ions inside the active material within the formed nanopores, favoring the hydration of the bulk oxides (Co_3_O_4_ and CdO) supported on the CdCO_3_ matrix, diminishing the charge transfer resistance. It also demonstrates that cadmium carbonate can serve as a support and give high stability to the electrode, as other studies have shown,^23^ since many of the electrodes for hybrid transition metal SCs need a support of graphene oxide or CNT in order to have high cycling stability. On assembling two SCs in series, and after charging them for only 10 s at 3 V, the device could efficiently power 5 mm blue (2.5 V, 2 mA) round light-emitting diode (LED) indicators, see inset of Fig. 6. For instance, a red LED remained very bright after 15 min as displayed in Fig. S8.† Moreover, these SCs can drive a mini rotation motor (operating current and voltage 8 mA and 0.18 V, respectively) strongly after 90 s of the charging step (see Movie in ESI†).

**Fig. 7:**
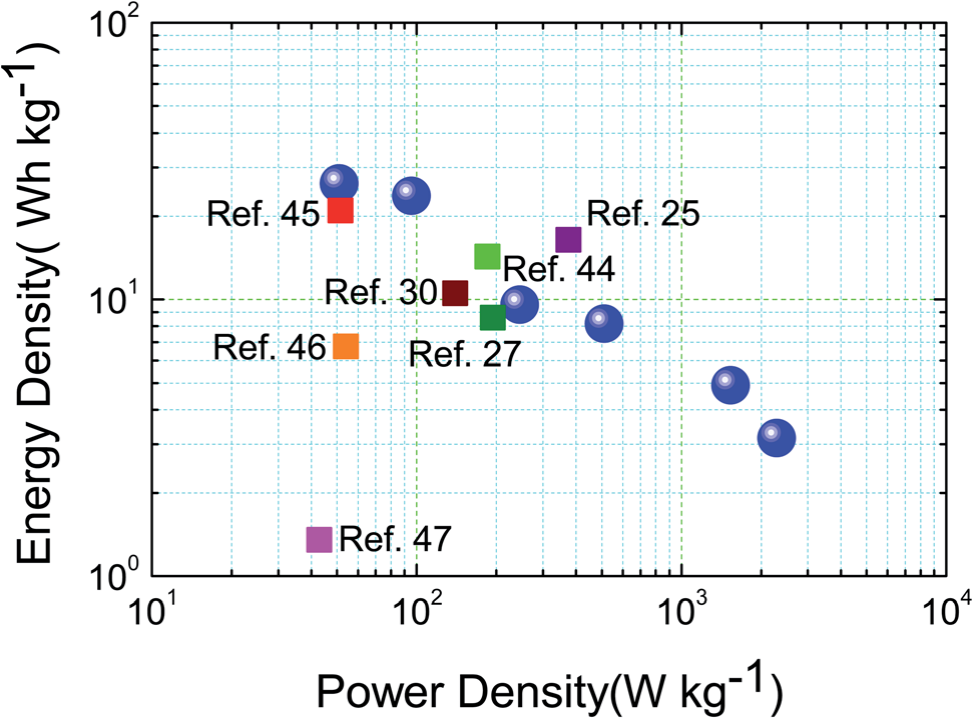
Ragone plot of the hybrid CdCO_3_/CdO/Co_3_O_4_@Ni//AC supercapacitor device (blue spheres), and the main reported values in the literature.

To demonstrate the overall electrochemical properties of the assembled device, the Ragone plot of the CdCO_3_/CdO/Co_3_O_4_@Ni//AC SC device (energy density (*E*) *vs.* power density (*P*)) is presented in Fig. 7, *E* and *P* values of the SC device were calculated from a double electrode system (see eqn (2) and (3)). Here, the SC device achieved an excellent energy density of 26.3 W h kg^−1^ at a power density of 51 W kg^−1^, which is comparable to, or even higher than, that of many previously reported supercapacitors such as CoO/Co_3_O_4_//AC,^30^ Co_3_O_4_//AC,^44^ MnCo_2_O_4_ based SC,^45^ PPy/Go/MnOx//AC,^46^ 3D rGO-based SC,^47^ MoS_2_/NiCo(OH)_2_CO_3_//AC^25^ and Co_3_O_4_@Co_2_(CO_3_)(OH)_2_//AC^27^ (see Fig. 7). This indicates the promising and practicable application of the CdCO_3_/CdO/Co_3_O_4_@Ni//AC SC device for energy storage fields.

## 4 Conclusions

A CdCO_3_/CdO/Co_3_O_4_@NF SC electrode has been prepared through direct growth without additives or binders *via* a facile hydrothermal-annealing method. This composite electrode has been effectively used as a positive electrode for hybrid SCs with an AC-based negative electrode. The as-assembled devices demonstrated a high specific capacitance of 84 F g^−1^ @ 1 mA cm^−2^ with a maximum energy density of 26.3 W h kg^−1^ and a power density of 2290 W kg^−1^ along with a widespread potential window of 1.5 V and long cycle life (92% after 6000 cycles). The as-assembled SCs (connected in series) were also employed to power various light-emitting diodes and move an electrical mini-motor. The excellent electrochemical properties demonstrate the promising potential applications of the Co_3_O_4_/CdO/CdCO_3_@NF in energy storage devices.

## Conflicts of interest

There are no conflicts to declare.

## Supplementary Material

RA-011-D1RA05243H-s001

RA-011-D1RA05243H-s002
