# Supplementary material for: High-performance asymmetric supercapacitor based on CdCO3/CdO/Co3O4 composite supported on Ni foam
Source: RSC Adv. 2021 Sep 23;11(50):31557–65. doi: 10.1039/d1ra05243h (PMC9041682; doi:10.1039/d1ra05243h)
Supplement: RA-011-D1RA05243H-s002 [file RA-011-D1RA05243H-s002.pdf]

Electronic Supplementary Material (ESI) for RSC Advances.
This journal is © The Royal Society of Chemistry 2021

Electronic Supporting Information (ESI)

High-performance asymmetric supercapacitor based on $CdCO_3$/CdO/Co_3O_4 composite supported on Ni foam

Rodrigo Henríquez,[*a] Alifhers S. Mestra-Acosta,[a] Eduardo Muñoz,[a] Paula Grez,[a] Elena Navarrete-Astorga[b] and Enrique A. Dalchiele[c]

[a.] Instituto de Química, Facultad de Ciencias, Pontificia Universidad Católica de Valparaíso, Casilla 4059, Valparaíso, Chile.

[b.] Universidad de Málaga, Departamento de Física Aplicada I, Laboratorio de Materiales y Superficies (Unidad asociada al CSIC), E29071 Málaga, Spain

[c.] Instituto de Física, Facultad de Ingeniería, Herrera y Reissig 565, C. C. 30, 11000 Montevideo, Uruguay.

A single asymmetric supercapacitor was first fully discharged at 0V at a discharge current of 50 mA cm^{-2} and then charged for 10s on a power source. It was then tested on a (Operating current 8mA and Operating voltage 0.18V- 0.6Vmin) mini fan which was in operation for a duration greater than 90s (see Video attached). **Two electrodes were also charged in series for 10s to turn on a 1 mA and 2.8V blue LED in a time of 0 to 3 min** and LED red of 1.8V for 0-15 min.

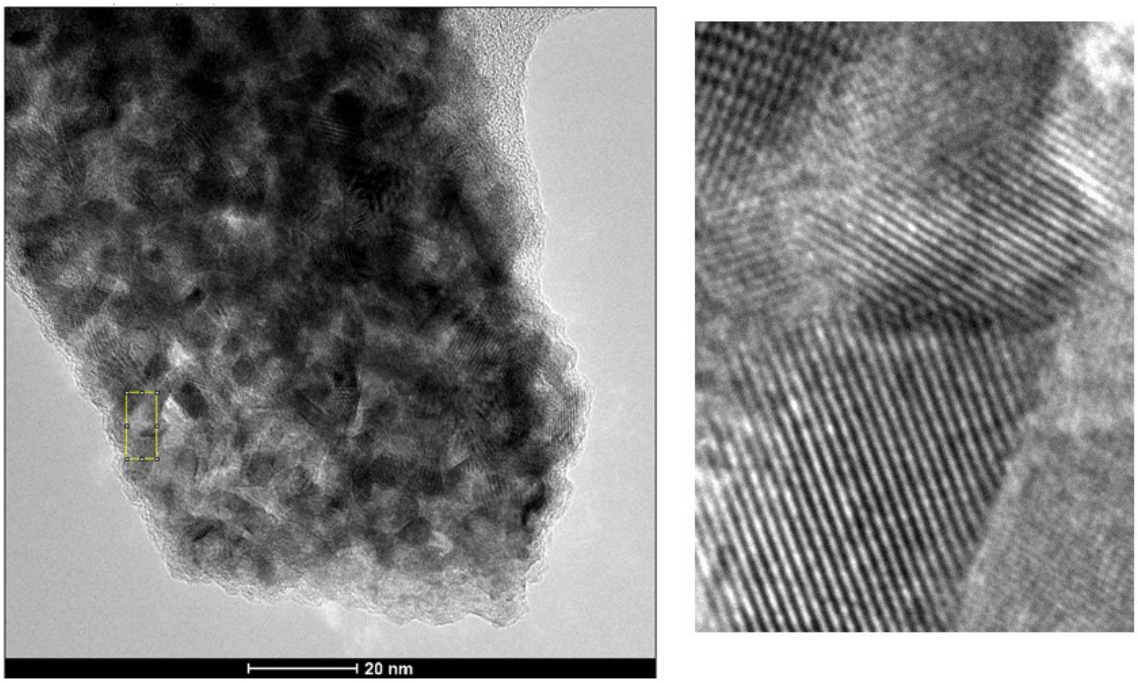

Figure S1. TEM images of the $CdCO_3/CdO/Co_3O_4$ composite after the annealing treatment under an argon atmosphere.

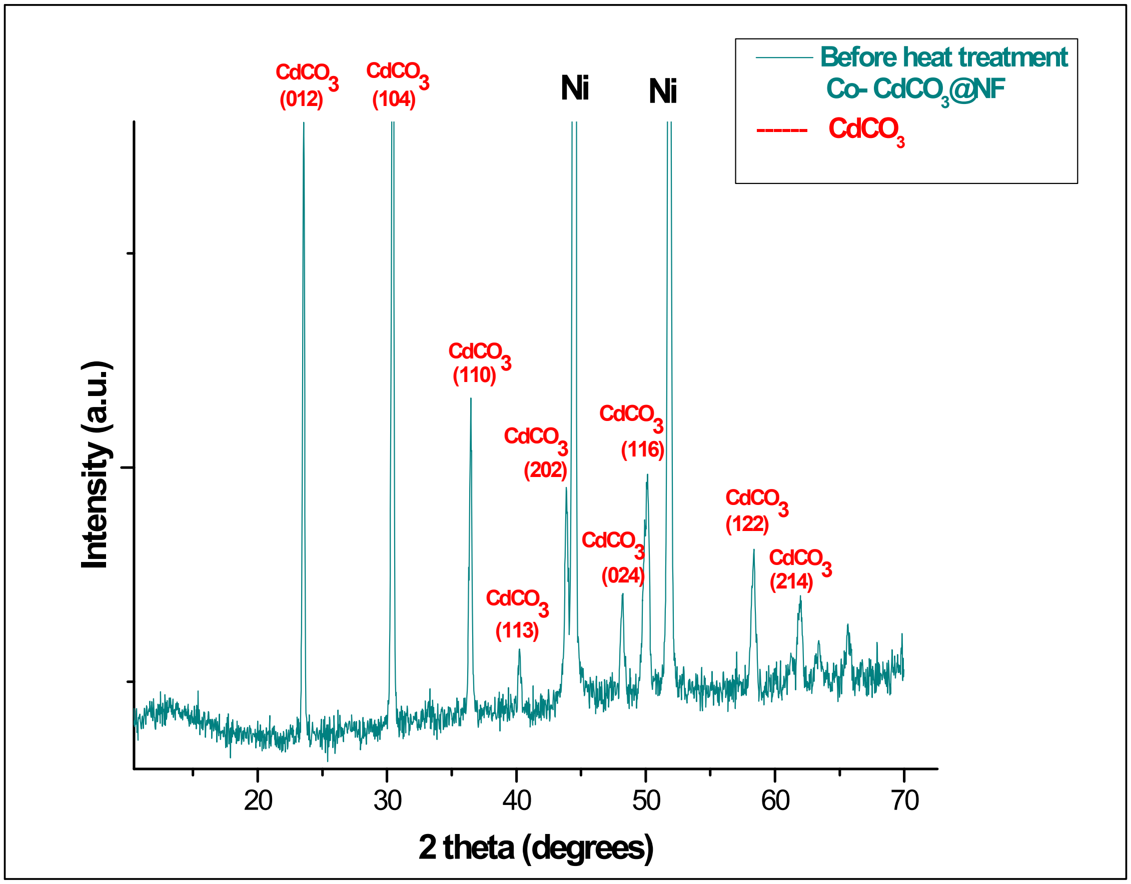

Figure S2. XRD diffraction pattern for a typical $CdCO_3/CdO/Co_3O_4$@NF sample, without the annealing process. Diffraction planes are indicated for the $CdCO_3$ phase. (Ni, indicates the peaks originated from the nickel foam substrate).

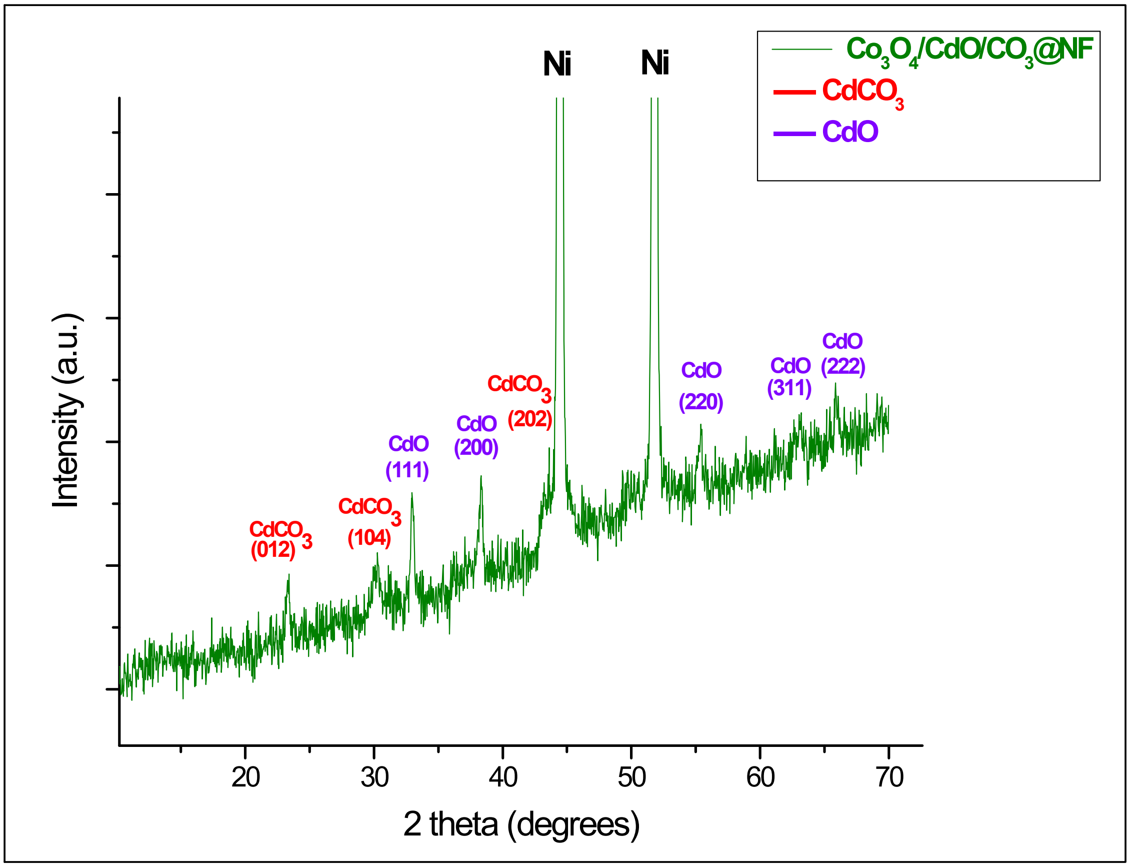

Figure S3. XRD diffraction pattern for a typical $CdCO_3/CdO/Co_3O_4$@NF electrode sample, after being submitted to the annealing process. Diffraction planes are indicated for the $CdCO_3$ and CdO phases. (Ni, indicates the peaks originated from the nickel foam substrate).

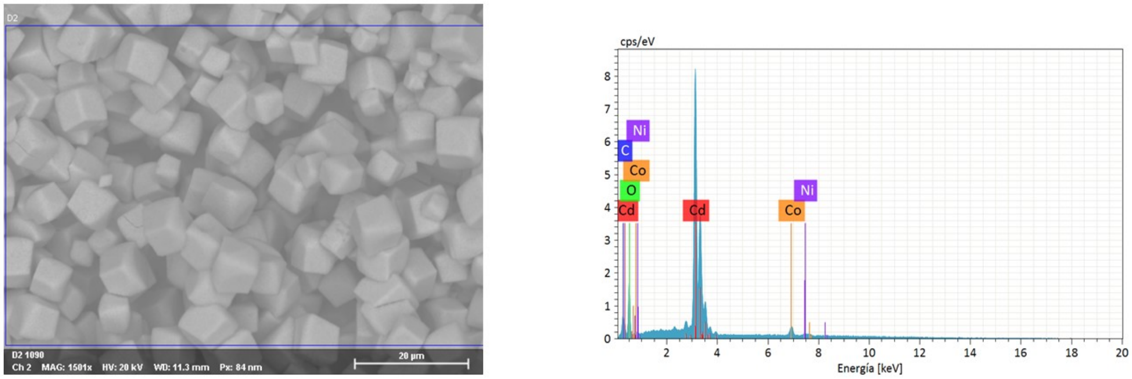

Figure S4. X-ray Dispersive Spectrometry (EDX) for the $CdCO_3/CdO/Co_3O_4$@NF composite electrode.

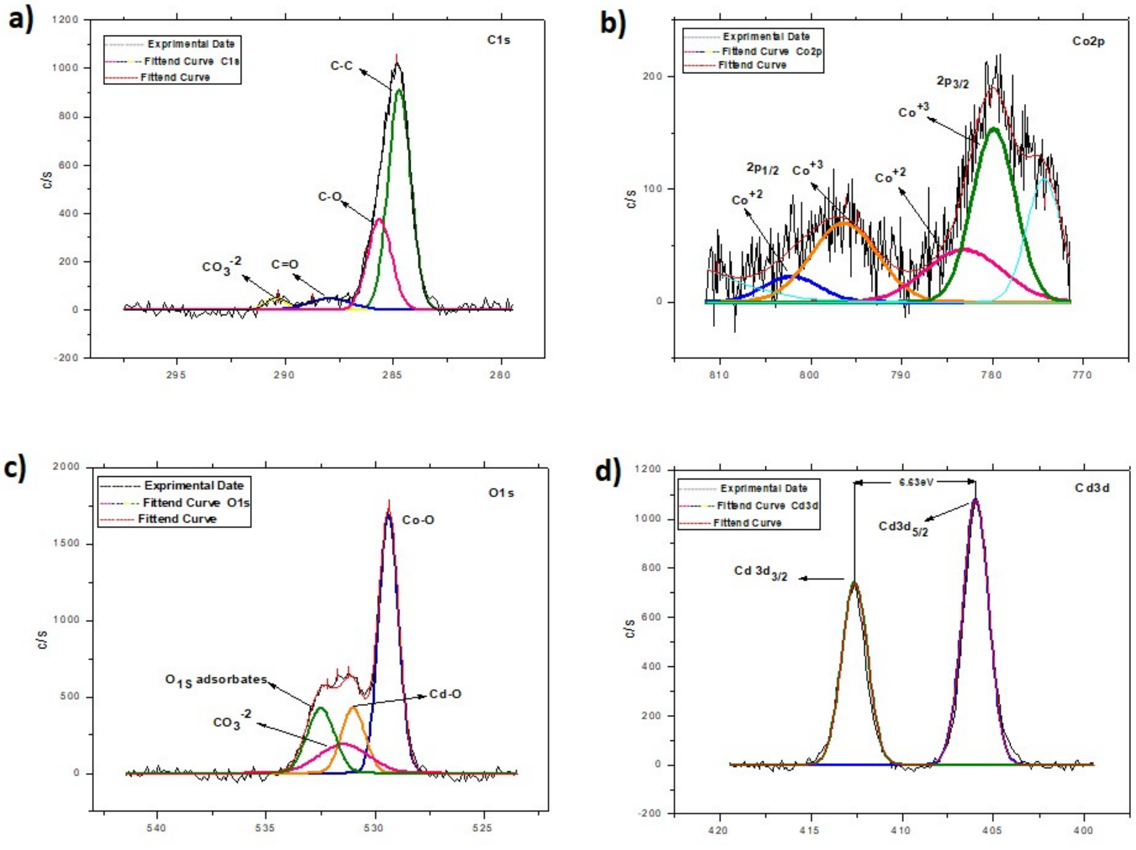

Figure S5. X-ray photoelectron spectroscopy analysis for the $CdCO_3/CdO/Co_3O_4$@NF composite electrode.

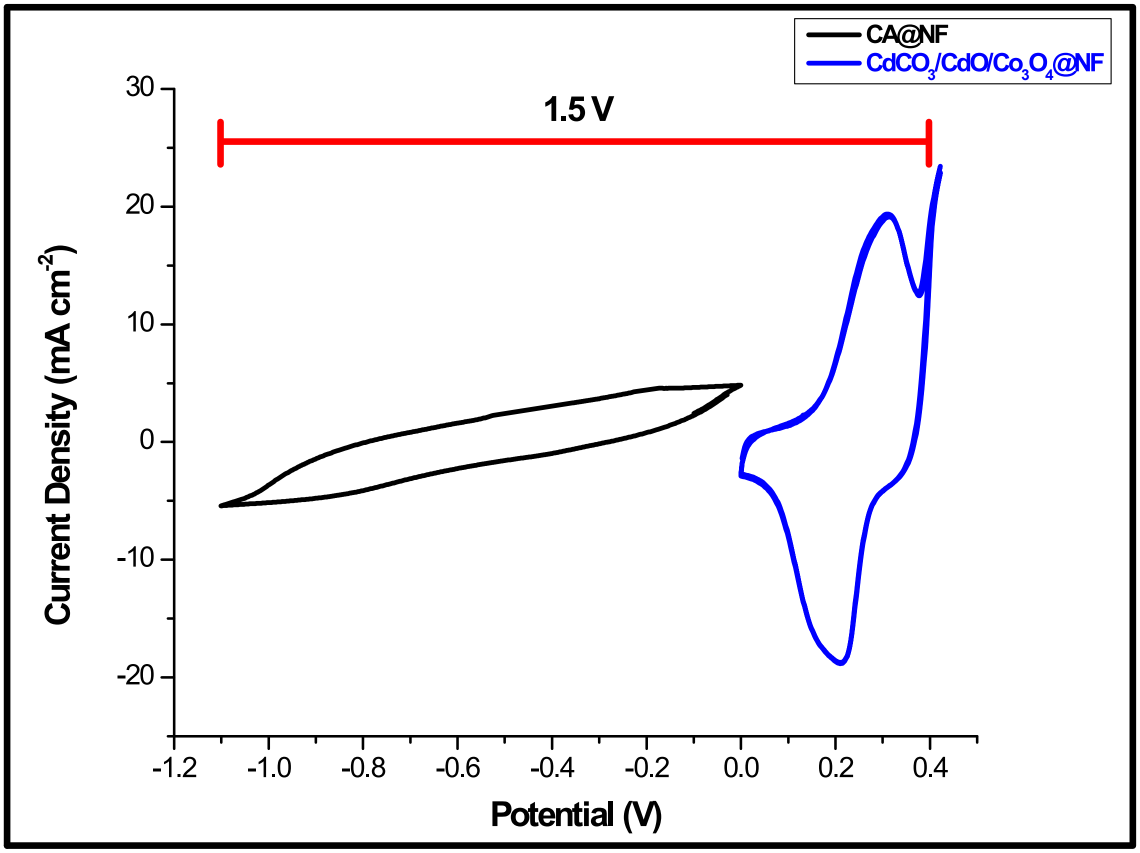

Figure S6. Cyclic voltammograms of the $CdCO_3/CdO/CO_3O_4$ @NF (blue line) and AC@NF (black line) electrodes, at a potential scan rate of 1 mVs^{-1}.

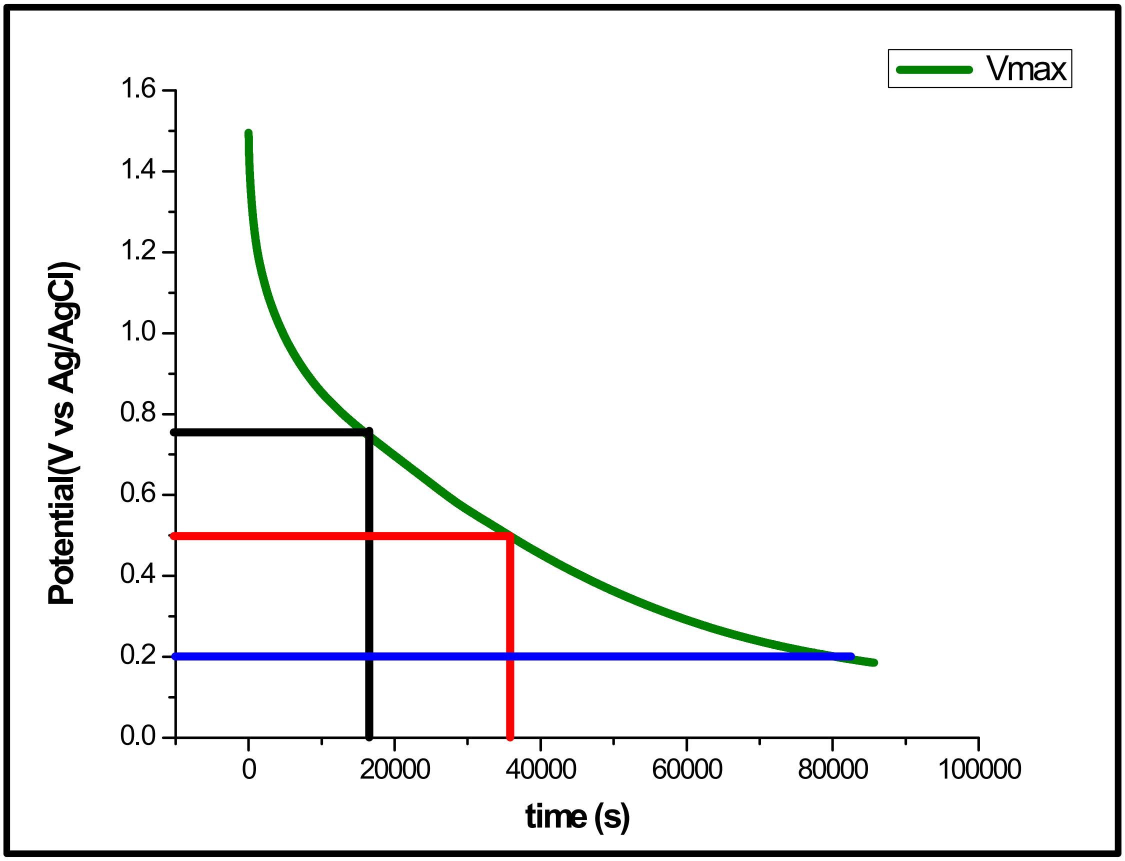

Figure. S7. Self-discharge curve of our supercapacitor device obtained immediately after pre-charge at Vmax in the previous test. This involves measuring the open circuit voltage across the supercapacitor between V_{max} and 1/2 V_{max} compared to the course of time. The result shows that our supercapacitor self-discharges from 1.5 V (V_{max}) to 0.75 V (1/2 V_{max}) in a prolonged time of 5h. The red line shows that the supercapacitor takes 10 hours to discharge to a voltage of 0.6V and the blue line indicates that after 24 hours the supercapacitor still has a small potential of 0.2V.

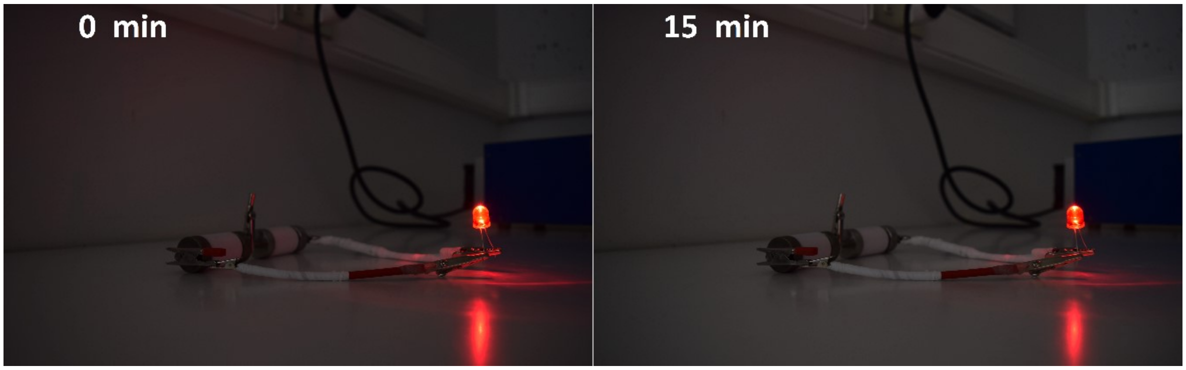

Figures S8. Red LED, operated by two as assembled supercapacitors in series at two different times after the charging step.

Table S1. Percentage of Cadmium, Oxygen, Cobalt, Carbon and Nickel obtained through the EDS analysis.

Element	At No	Netto	Mas [%]	Mass Norm [%]	Atom [%]
**Cd**	48	45125	75.63	70.89	27.15
**O**	8	3271	23.00	21.56	58.00
**Co**	27	1378	3.62	3.39	2.48
**C**	6	1029	3.49	3.27	11.72
**Ni**	28	365	0.95	0.89	0.65
	Sum	106.69	100.00	100.00	

Table S2. Specific capacitance of the asymmetric hybrid supercapacitor at different potential scan rates.

**V(mVs^{-1})**	**Q(A.s)**	**E(V)**	**m(g)**	**C(Fg^{-1})**
5	1.70	1.5	0.023	24.66
10	1.45	1.5	0.023	21.10
20	1.17	1.5	0.023	17.08
30	1.01	1.5	0.023	14.73
50	0.81	1.5	0.023	11.86

Table S3. Specific capacitance, energy density and power densities of the asymmetric hybrid supercapacitor at different current densities.

Chage/ Descharge Current (mA cm^{-2})	**1**	**2**	**5**	**10**	**30**	**50**
Specific Capacitance (Fg^{-1})	84.14	81.45	30.60	26.14	15.68	11.36
Energy Density (Wh kg^{-1})	26.29	23.73	9.56	8.17	4.9	3.16
Power Density (W kg^{-1})	51	95.68	255.88	511.42	1536	2289.65
